# Acute effect of Finnish sauna bathing on brachial artery flow‐mediated dilation and reactive hyperemia in healthy middle‐aged and older adults

**DOI:** 10.14814/phy2.14166

**Published:** 2019-07-10

**Authors:** Hugo Gravel, Geoff B. Coombs, Parya Behzadi, Virginie Marcoux‐Clément, Hadiatou Barry, Martin Juneau, Anil Nigam, Daniel Gagnon

**Affiliations:** ^1^ Cardiovascular Prevention and Rehabilitation Centre Montreal Heart Institute Montréal Canada; ^2^ Département de Pharmacologie et Physiologie Université de Montréal Montréal Canada; ^3^ School of Health and Exercise Sciences University of British Columbia – Okanagan Kelowna Canada

**Keywords:** Aging, flow‐mediated dilation, heat

## Abstract

Regular Finnish sauna bathing is associated with a reduced risk of all‐cause and cardiovascular mortality in middle‐aged and older adults. Potential acute physiological adaptations induced by sauna bathing that underlie this relationship remain to be fully elucidated. The purpose of this study was to determine if typical Finnish sauna sessions acutely improve brachial artery flow‐mediated dilation (FMD) and reactive hyperemia (RH) in healthy middle‐aged and older adults. Using a randomized crossover design, FMD and RH were evaluated in 21 healthy adults (66 ± 6 years, 10 men/11 women) before and after each of the following conditions: (1) 1 × 10 min of Finnish sauna bathing (80.2 ± 3.2°C, 23 ± 2% humidity); (2) 2 × 10 min of sauna bathing separated by 10 min of rest outside the sauna; (3) a time control period (10 min of seated rest outside the sauna). FMD was taken as the peak change from baseline in brachial artery diameter following 5 min of forearm ischemia, whereas RH was quantified as both peak and area‐under‐the‐curve forearm vascular conductance postischemia. FMD was statistically similar pre to post 1 × 10 min (4.69 ± 2.46 to 5.41 ± 2.64%, *P *= 0.20) and 2 × 10 min of sauna bathing (4.16 ± 1.79 to 4.55 ± 2.14%, *P *= 0.58). Peak and area‐under‐the‐curve forearm vascular conductance were also similar following both sauna interventions. These results suggest that typical Finnish sauna bathing sessions do not acutely improve brachial artery FMD and RH in healthy middle‐aged and older adults.

## Introduction

Recent analyses of the Kuopio Ischemic Heart Disease risk factor study provide compelling evidence for the long‐term health benefits of Finnish sauna bathing (Laukkanen et al. 2018). In a large cohort of middle‐aged Finnish males, greater frequency (≥2–3 times per week) and greater duration (>19 min per session) of sauna use were associated with a decreased risk of sudden cardiac death, cardiovascular and all‐cause mortality (Laukkanen et al. 2015), hypertension (Zaccardi et al. 2017), stroke (Kunutsor et al. 2018), as well as dementia and Alzheimer's disease (Laukkanen et al. 2017). Given the study cohort spanned the health continuum (healthy, at‐risk, overt disease), the results suggest that Finnish sauna bathing may represent a recommendable lifestyle habit for primary and/or secondary prevention of cardiovascular diseases. However, potential physiological adaptations linking Finnish sauna use with these long‐term health benefits remain to be elucidated.

Acute physiological responses elicited by Finnish sauna bathing may lead to chronic adaptations that reduce the risk of adverse cardiovascular events in long‐term sauna users. Studies have demonstrated that Finnish sauna bathing acutely decreases arterial blood pressure and arterial stiffness (Gayda et al. 2012; Radtke et al. 2016; Lee et al. 2018). In addition to such responses, improved vascular health may contribute to the long‐term cardiovascular benefits of Finnish sauna bathing. It is becoming increasingly recognized that the cardiovascular responses associated with heat exposure exert positive effects upon markers of vascular health (Cheng and MacDonald 2019). Notably, studies have demonstrated that acute (Tinken et al. 2009; Romero et al. 2017) and repeated (Imamura et al. 2001; Kihara et al. 2002; Carter et al. 2014; Brunt et al. 2016b) heat exposure improves flow‐mediated dilation and/or reactive hyperemia, two independent markers of future cardiovascular events (Yeboah et al. 2009; Anderson et al. 2011; Matsuzawa et al. 2015; Cooper et al. 2016). However, studies performed thus far have employed varying passive heating modalities other than Finnish sauna bathing (infrared sauna, hot water immersion, etc.) and most have employed relatively large “doses” of heat exposure (e.g. water immersion at 40–41°C for 30–90 min). In contrast, typical Finnish sauna bathing consists of brief (5–20 min) exposures in dry heat (70–100°C, 10–20% relative humidity) separated by rest or cooling periods outside of the sauna (Heinonen and Laukkanen 2018; Laukkanen et al. 2018). Although Finnish sauna bathing increases skin perfusion, heart rate, cardiac output, and internal body temperature (Smolander and Kolari 1985; Kauppinen 1989a,1989b), it remains unknown if it provides enough of a stimulus to acutely improve flow‐mediated dilation and/or reactive hyperemia. The aim of the current study was to test the hypothesis that brachial artery flow‐mediated dilation and reactive hyperemia are acutely augmented by typical Finnish sauna bathing sessions in healthy middle‐aged and older adults.

## Methods

### Ethical approval

This study was approved by the Montreal Heart Institute Research Ethics and New Technology Development Committee (#2017–2179). All participants provided written informed consent prior to their participation in the study.

### Participants

Twenty‐one participants, 50–80 years of age, were recruited for the study. All participants were healthy, free of overt cardiovascular diseases and not taking medication for the primary or secondary prevention of cardiovascular diseases. Women participants were postmenopausal and not on hormonal replacement therapy. Exclusion criteria were: a diagnosis of or pharmacological treatment for hypertension, diabetes, dyslipidemia; resting systolic blood pressure >140 mmHg and/or diastolic blood pressure >90 mmHg; history of smoking in the preceding 5 years. Eligibility was determined during preliminary visits with detailed medical history and lifestyle questionnaires, a resting (≥10 min in supine position) 12‐lead ECG and blood pressure measurement and a fasting (≥12 h) blood sample. Physical activity level and sedentary time were assessed using the Global Physical Activity Questionnaire (Cleland et al. 2014). Eight of the 21 participants reported that they used saunas at least once per week. For these participants, mean frequency of sauna use was 2.5 days/week (range: 1–3 days/week), and the mean weekly sauna duration was 34 min/week (range: 5–66 min/week).

### Experimental protocol

The study design was a crossover trial involving 3 experimental visits performed in random sequence. All visits took place between October and July and started between 7:30 and 9:30 am. During each visit, flow‐mediated dilation and reactive hyperemia were evaluated before and after one of the following conditions: (1) 1 × 10 min of sauna bathing (1 × 10); (2) 2 × 10 min of sauna bathing separated by 10 min of rest outside the sauna (2 × 10), or; (3) a time control period consisting of 10 min of seated rest outside the sauna. The sauna interventions were chosen to reflect typical Finnish sauna bathing practice (Heinonen and Laukkanen 2018; Laukkanen et al. 2018). In addition, the durations chosen were consistent with the mean session duration (~14 min) reported for groups of regular Finnish sauna users (2–3 and 4–7 times per week) who demonstrated a reduced risk of all‐cause and cardiovascular mortality (Laukkanen et al. 2015). The experimental visits were scheduled ≥48 h apart. Before each visit, participants were asked to fast for 12 h and consume a standardized snack (47 g carbohydrate, 3.5 g fat, 2 g protein) 1 h prior to their arrival to the laboratory. Participants were also asked to refrain from alcohol and caffeine or other stimulants, and to avoid intense physical activity and heat exposure for 12 h prior to each visit.

Upon arrival to the laboratory, participants provided a urine sample to quantify hydration status before weighing themselves nude. They then dressed into shorts (for men) or shorts and a sports bra or loose t‐shirt (for women) and were instrumented before lying down supine within a quiet and thermoneutral environment (24°C). After 30 min of supine rest, flow‐mediated dilation and reactive hyperemia were evaluated. Participants then walked to the sauna and sat upright outside the sauna for a minimum of 5 min to obtain pre‐sauna measurements of heart rate, blood pressure, and body temperatures. Participants were subsequently exposed to one of the 3 experimental conditions, after which they walked back to the laboratory where flow‐mediated dilation and reactive hyperemia were reassessed following 30 min of supine rest. A 30‐min recovery period was chosen to allow brachial artery diameter to return to baseline values. For the conditions involving sauna bathing, participants sat upright on the upper bench of a traditional Finnish sauna maintained at 80.2 ± 3.2°C and 23 ± 2% relative humidity. For the condition involving 2 sauna exposures, participants sat upright outside the sauna during the 10‐min rest period. Participants could drink water ad libitum during the experimental visits. A final nude weight was obtained at the end of the visit.

### Measurements

Body height was measured with a stadiometer (model 216, Seca) and body mass with a high‐performance digital scale (IND236, Mettler‐Toledo, precision: 0.01 kg). Urine specific gravity was measured as an index of hydration status with a digital refractometer (PAL‐10S, Atago). Heart rate was obtained from lead II of a continuous 5‐lead low‐pass filtered ECG signal (Solar i8000, GE Healthcare). Systolic and diastolic blood pressures were measured by ECG‐gated automatic auscultation of the brachial artery (Tango M2, SunTech Medical). Skin temperatures were measured at the chest, shoulder, thigh, and calf with wireless sensors (iButtons, Embedded Data Systems) taped to the skin surface. Oral temperature was measured with a handheld thermometer (SureTemp Plus 690, Welch Allyn).

Brachial artery flow‐mediated dilation (FMD) was measured according to the most recent guidelines (Thijssen et al. 2011). Brachial artery diameter and peak blood velocity were measured simultaneously by high‐resolution Doppler ultrasound (uSmart3300, Terason) equipped with a 4‐15 MHz linear array transducer probe at an insonation angle of 60°. The participants rested supine with their right arm supported at 75–90° abduction. A rapid inflation/deflation pneumatic cuff (SC5, Hokanson) was placed immediately distal to the antecubital fossa. The ultrasound probe was placed 5–15 cm proximal to the right antecubital fossa, where an optimal B‐mode image could be obtained. The measurement site was identified with a surgical marker and successive measurements in a given participant were made at the same site. A baseline recording of brachial artery diameter and blood velocity was performed for 1 min following which the forearm cuff was inflated to 250 mmHg for 5 min by a rapid cuff inflator (E20, Hokanson). The recording resumed 1 min before cuff deflation and continued for 3 min postdeflation. Ultrasound recordings were sent to a remote computer using a frame grabber (DVIUSB 3.0, Epiphan), were video captured (Camtasia v.9, TechSmith) and later analyzed, using edge‐detection and wall‐tracking software (Cardiovascular Suite v.3, Quipu SRL). This method provided measurements of arterial diameter, as well as time‐averaged positive (antegrade) and negative (retrograde) blood velocities based on the Doppler envelope, at a sampling rate of 30 Hz.

### Data analyses

Brachial artery diameter and blood velocities were averaged in successive 1‐sec bins. Baseline brachial artery diameter (*D*_base_) was defined as the average diameter during the 1‐min baseline recording. Peak brachial artery diameter (*D*_peak_) was defined as the maximal 1‐sec average observed during the postocclusion period. Unadjusted FMD was determined as the percentage change in brachial artery diameter from baseline to peak: (*D*_peak_ − *D*_base_)/*D*_base_ × 100%. Shear rate area‐under‐the‐curve (SR_AUC_) up to peak diameter was considered the stimulus for FMD. Shear rate was calculated as: 4 × mean blood velocity/diameter. Antegrade and retrograde shear rates were calculated using positive and negative mean blood velocity values, respectively. Reactive hyperemia was quantified as peak and area‐under‐the‐curve (AUC) forearm vascular conductance during the 3‐min postocclusion period. Forearm vascular conductance was calculated as forearm blood flow divided by the mean arterial pressure measured immediately before each FMD assessment. Forearm blood flow was calculated as vessel cross‐sectional area multiplied by mean blood velocity. Mean arterial pressure was calculated as diastolic pressure +1/3 of pulse pressure. Mean skin temperature was calculated as a weighted summation of the four skin temperature measurements using the formula (Ramanathan 1964): 0.3 × (upper arm + chest) + 0.2 × (thigh + calf). Total sweat loss was calculated as the difference in nude body weight measurements corrected for any fluid intake and/or urine output.

### Statistical analyses

Based on a previous study that reported a mean ± standard deviation increase in superficial femoral artery FMD of 1.5 ± 1.5% following 45 min of lower limb heating in healthy older adults (Romero et al. 2017), we calculated a priori that 16 participants would provide 80% power to detect a 1.5 ± 1.5% change in FMD using a two‐tailed paired sample t‐test with a level of significance of 0.01. A 1.5% increase in brachial artery FMD was considered meaningful, as the relative risk of cardiovascular events decreases by 13% per 1% increase in FMD (Inaba et al. 2010). The Shapiro–Wilk test was used to verify normality of data distribution at each time point. For normal distributions, statistical analyses were performed using paired t‐tests for data measured at 2 time points and repeated measures one‐way ANOVA for data measured at multiple time points, using Greenhouse–Geisser's adjustment for degrees of freedom when there was deviation from the assumption of sphericity, as detected by Mauchly's test. When distributions deviated from normality at one or more time points, Wilcoxon signed‐ranks test was used to compare variables measured at two time points and Friedman test was used to compare data measured at multiple time points. When the ANOVA or Friedman test detected a significant main effect, pairwise comparisons were conducted using Sidak's adjustment. FMD measurements of sufficient quality for analysis were obtained in 16, 16, and 19 participants during the control, 1 × 10, and 2 × 10 conditions, respectively. Therefore, statistical analyses were performed for each condition separately. To account for potential *D*_base_ variations between repeated FMD measurements, a linear mixed model was used to compare the main outcome of «log *D*_peak_ − log *D*_base_» between pre and postsauna or time control, while using log *D*_base_ as a covariate (Atkinson and Batterham 2013). SR_AUC_ was also included as a covariate in the model to account for potential changes in the stimulus for FMD between repeated measurements. The critical *P* value was set at <0.05. Statistical analyses were performed using IBM SPSS v.24. All data are reported as mean ± standard deviation. The change in unadjusted FMD and reactive hyperemia during each condition is also presented as mean [95% confidence intervals].

## Results

Participant characteristics are reported in Table 1. Urine specific gravity upon arrival to the laboratory was similar between conditions (1.015 ± 0.006, 1.015 ± 0.006 and 1.017 ± 0.007 in the control, 1 × 10 and 2 × 10 conditions, respectively, *P *=* *0.69).

**Table 1:** Participant characteristics

Variable	Value
Men/Women	10/11
Age (years)	66 ± 7 (51–79)
Body mass index (kg/m^2^)	26.3 ± 2.5 (21.5–30.3)
Resting heart rate (bpm)	60 ± 9 (44–75)
Systolic blood pressure (mmHg)	122 ± 10 (103–139)
Diastolic blood pressure (mmHg)	77 ± 8 (62–90)
Fasting glucose (mmol/L)	5.0 ± 0.5 (4.4–6.1)
Hb‐A1C (%)	5.5 ± 0.3 (5.0–6.4)
Total cholesterol (mmol/L)	5.2 ± 0.7 (3.8–6.3)
Low‐density lipoprotein (mmol/L)	2.8 ± 0.7 (1.5–3.9)
High‐density lipoprotein (mmol/L)	1.9 ± 0.5 (1.4–3.0)
Triglycerides (mmol/L)	1.2 ± 0.5 (0.5–2.6)
Physical activity level (METs*min/week)	3106 ± 1731 (720–6480)
Sedentary time (h/d)	7.3 ± 3.9 (2.0–16.0)

Values are mean ± SD (range).

### Sauna exposure

All participants completed the 1 × 10 sauna session. All but one participant completed the 2 × 10 sauna session; one participant exited the sauna after 9 min of the second sauna bath due to hypotension (<90/60 mmHg). Sauna bathing increased mean skin temperature, oral temperature, and heart rate, whereas it decreased systolic, diastolic, and mean blood pressures (Table 2). Sweat loss was lower during the control condition (0.13 ± 0.07 kg) than during the 1 × 10 (0.21 ± 0.08 kg) and 2 × 10 conditions (0.35 ± 0.11 kg, *P *≤* *0.01 for pairwise comparisons).

**Table 2:** Body temperatures, heart rate and arterial blood pressure during the 3 experimental conditions

		Responses during the interventions					Values at time of vascular testing		
		Baseline	Sauna 1	Recovery	Sauna 2	*P*‐value	PRE	POST	*P*‐value
Mean skin temperature (°C)	Control	–	–	–	–	–	32.8 ± 0.5	33.0 ± 0.7	0.45
	1 × 10	33.0 ± 0.5	41.5 ± 0.6	–	–	<0.01	33.0 ± 0.5	34.6 ± 0.5	<0.01
	2 × 10	32.9 ± 0.5	40.9 ± 1.1*	35.0 ± 0.6*	41.2 ± 0.9*	<0.01	32.9 ± 0.5	34.2 ± 0.8	<0.01
Oral temperature (°C)	Control	–	–	–	–	–	36.7 ± 0.2	36.6 ± 0.2	0.11
	1 × 10	36.6 ± 0.2	37.1 ± 0.2	–	–	<0.01	36.6 ± 0.2	36.7 ± 0.2	0.10
	2 × 10	36.5 ± 0.2	37.1 ± 0.2*	37.0 ± 0.1*	37.4 ± 0.3*	<0.01	36.7 ± 0.2	36.8 ± 0.1	0.02
Heart rate (bpm)	Control	–	–	–	–	–	55 ± 6	53 ± 7	0.07
	1 × 10	58 ± 10	81 ± 14	–	–	<0.01	56 ± 9	58 ± 10	0.04
	2 × 10	57 ± 8	81 ± 12*	67 ± 10*	90 ± 13*	<0.01	55 ± 6	58 ± 8	0.02
Systolic blood pressure (mmHg)	Control	–	–	–	–	–	117 ± 12	123 ± 16	0.06
	1 × 10	123 ± 15	105 ± 11	–	–	<0.01	117 ± 12	115 ± 9	0.30
	2 × 10	124 ± 14	111 ± 12*	117 ± 16	107 ± 18*	<0.01	116 ± 11	116 ± 9	0.70
Diastolic blood pressure (mmHg)	Control	–	–	–	–	–	73 ± 9	76 ± 9	0.17
	1 × 10	78 ± 7	72 ± 12	–	–	0.03	74 ± 7	74 ± 7	0.68
	2 × 10	78 ± 10	75 ± 9	80 ± 9	74 ± 13	0.01	74 ± 9	74 ± 9	0.82
Mean arterial pressure (mmHg)	Control	–	–	–	–	–	88 ± 9	92 ± 9	0.04
	1 × 10	93 ± 9	83 ± 10*	–	–	<0.01	89 ± 7	88 ± 7	0.39
	2 × 10	93 ± 11	87 ± 9*	92 ± 10	85 ± 13*	<0.01	88 ± 8	88 ± 8	0.71

Data are mean ± SD for *n* = 21. *P < 0.05 versus baseline (Sidak's adjustment).

### Effect of sauna bathing on FMD and reactive hyperemia

Transition time between the end of sauna bathing and the start of the 30‐min postsauna period was 9.3 ± 4.3 min. Body temperatures, heart rate and blood pressure measured at the time of flow‐mediated dilation and reactive hyperemia assessments are reported in Table 2. Systolic and diastolic blood pressure was similar at the time of pre‐ and postmeasurements during all conditions, whereas mean arterial pressure was statistically elevated during the postmeasurement in the control condition. Heart rate and body temperatures were similar at the time of pre‐ and postassessments during the control condition. However, mean skin temperature and heart rate remained elevated for postsauna assessments during the 1 × 10 and 2 × 10 conditions, and oral temperature remained elevated postsauna during the 2 × 10 condition.

**Figure 1:**
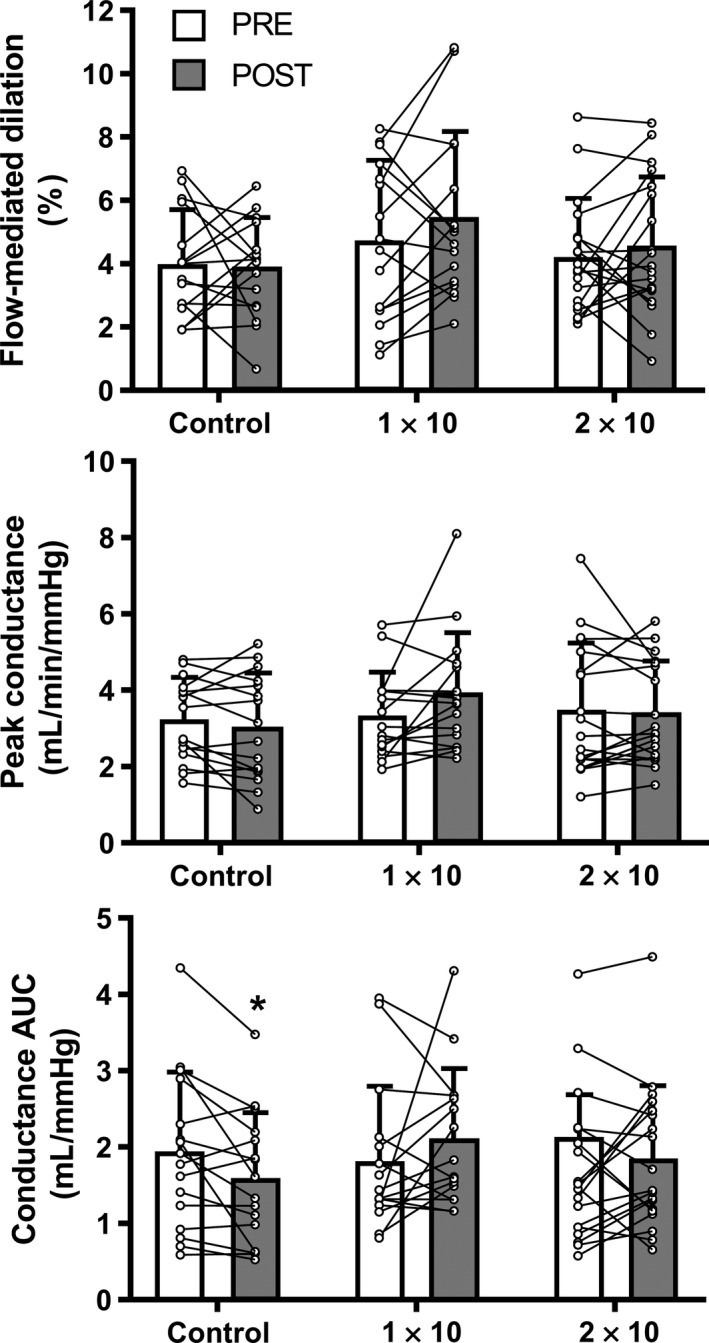
Brachial artery flow‐mediated dilation (FMD, unadjusted), peak and area‐under‐the‐curve (AUC) forearm vascular conductance during reactive hyperemia before (PRE) and after (POST) sauna or control interventions. Boxes and error bars show means and standard deviations. Dots show individual values obtained for *n* = 16 (control), *n* = 16 (1 × 10) and *n* = 19 (2 × 10). Data were compared between PRE and POST by paired *t*‐tests. *P < 0.05 versus PRE within condition.

Before cuff inflation, brachial artery diameter was similar during the pre‐ and postassessments during all conditions. Baseline shear pattern was similar before and after the control intervention (Table 3). Antegrade shear rate was increased and retrograde shear rate decreased postsauna during the 1 × 10, but not 2 × 10, condition (Table 3). Similarly, baseline forearm blood flow and vascular conductance were greater before the postmeasurement during the 1 × 10, but not control or 2 × 10 conditions (Table 3). SR_AUC_ following cuff deflation was similar before and after the control and 2 × 10 conditions but was increased postsauna during the 1 × 10 condition (Table 3). Time to peak dilation of the brachial artery was similar before and after the control intervention but was increased after both sauna conditions (Table 3). The pre‐ to postintervention difference in unadjusted FMD was −0.11% [−1.14, 0.92], 0.72% [−0.38, 1.82] and 0.38% [−0.50, 1.27] during the control, 1 × 10, and 2 × 10 conditions, respectively. Within each condition, the pre‐ and postintervention unadjusted FMD values were statistically similar (*P *>* *0.18 for each condition, Fig. 1). When adjusting for *D*_base_ and SR_AUC,_ FMD was also statistically similar between pre‐ and postintervention during the control (*P *=* *0.95), 1 × 10 (*P *=* *0.20), and 2 × 10 (*P *=* *0.58) conditions (data not presented).

**Table 3:** Flow‐mediated dilation variables before (PRE) and after (POST) sauna or control interventions

Variable	Condition	PRE	POST	*P*‐value
Baseline brachial artery diameter (mm)	Control	4.12 ± 0.75	4.12 ± 0.70	0.98
	1 × 10	4.08 ± 0.74	4.13 ± 0.73	0.28
	2 × 10	4.10 ± 0.82	4.10 ± 0.77	0.99
Baseline forearm blood flow (mL/min)	Control	55 ± 37	47 ± 27	0.23
	1 × 10	71 ± 44	110 ± 53	<0.01
	2 × 10	83 ± 75	79 ± 57	0.94
Baseline forearm vascular conductance (mL/min/mmHg)	Control	0.63 ± 0.39	0.53 ± 0.32	0.17
	1 × 10	0.81 ± 0.48	1.27 ± 0.64	<0.01
	2 × 10	0.94 ± 0.85	0.91 ± 0.70	0.78
Baseline antegrade shear rate (sec^−1^)	Control	75 ± 30	65 ± 31	0.22
	1 × 10	103 ± 59	134 ± 48	<0.01
	2 × 10	92 ± 43	97 ± 36	0.62
Baseline retrograde shear rate (sec^−1^)	Control	7 ± 7	8 ± 7	0.96
	1 × 10	8 ± 8	2 ± 2	<0.01
	2 × 10	6 ± 7	4 ± 4	0.33
Peak FMD dilation (mm)	Control	0.16 ± 0.08	0.16 ± 0.07	0.96
	1 × 10	0.18 ± 0.09	0.22 ± 0.09	0.12
	2 × 10	0.16 ± 0.06	0.18 ± 0.07	0.34
Time to peak dilation (ecs)	Control	51 ± 26	44 ± 13	0.52
	1 × 10	50 ± 16	68 ± 21	0.01
	2 × 10	51 ± 23	60 ± 13	0.03
SR_AUC_ to peak (a.u.)	Control	7.3 ± 2.0	6.5 ± 2.5	0.23
	1 × 10	7.6 ± 3.1	9.0 ± 3.5	0.05
	2 × 10	7.5 ± 3.5	8.6 ± 3.5	0.20

Data are mean ± SD for *n* = 16 (control), *n* = 16 (1 × 10) and *n* = 19 (2 × 10). FMD, flow‐mediated dilation; SR_AUC_, Shear rate area‐under‐the‐curve.

From pre‐ to postintervention, peak reactive hyperemia changed by −0.19 mL/min/mmHg [−0.55, 0.17], 0.60 mL/min/mmHg [−0.06, 1.26] and −0.07 mL/min/mmHg [−0.50, 0.35] during the control, 1 × 10, and 2 × 10 conditions, respectively. Peak forearm vascular conductance during reactive hyperaemia was statistically similar between pre‐ and postassessments for all conditions (*P *>* *0.07 for each condition, Fig. 1). When expressed as AUC, reactive hyperemia changed by −0.35 mL/mmHg [−0.64, −0.06], 0.29 mL/mmHg [−0.23, 0.81], 0.14 mL/mmHg [−0.18, 0.46] during the control, 1 × 10, and 2 × 10 conditions, respectively. Statistically, forearm vascular conductance AUC decreased after the time control period (*P *=* *0.02), whereas it was similar during pre‐ and postsauna assessments for the 1 × 10 (*P *=* *0.25) and 2 × 10 (*P *=* *0.37) conditions (Fig. 1).

## Discussion

The current study evaluated the acute effect of typical Finnish sauna bathing sessions on brachial artery flow‐mediated dilation and forearm reactive hyperemia in healthy middle‐aged and older adults. Shorter (10 min) and longer (20 min) Finnish sauna sessions did not affect brachial artery flow‐mediated dilation, as well as peak and area‐under‐the‐curve forearm vascular conductance during reactive hyperemia. These data suggest that typical Finnish sauna bathing sessions do not acutely improve brachial artery flow‐mediated dilation and reactive hyperemia in healthy middle‐aged and older adults.

Recent studies suggest that Finnish sauna bathing may represent a lifestyle intervention that is particularly beneficial for the primary and/or secondary prevention of cardiovascular diseases. Specifically, middle‐aged Finnish men who self‐reported using sauna 4–7 times per week (~14 min per session) or reported sauna durations >19 min had a 24% to 60% risk reduction for sudden cardiac death, fatal coronary disease and fatal cardiovascular disease over a 20‐year follow‐up (Laukkanen et al. 2015). Although these relationships were independent of several confounding variables, potential physiological adaptations suggesting a direct link between Finnish sauna bathing and improved cardiovascular health remain to be fully established. Presumably, the long‐term benefits of Finnish sauna bathing may stem from the accumulation of acute physiological adaptations to sauna bathing. Accordingly, studies have demonstrated that Finnish sauna bathing acutely lowers blood pressure and arterial stiffness (Gayda et al. 2012; Radtke et al. 2016; Lee et al. 2018). Considering that brachial artery flow‐mediated dilation (Yeboah et al. 2009; Matsuzawa et al. 2015) and peak reactive hyperemia (Anderson et al. 2011; Cooper et al. 2016) independently predict cardiovascular events in the general population, we hypothesized that Finnish sauna bathing would result in acute improvements in these markers of vascular health. Contrary to our hypothesis, flow‐mediated dilation and reactive hyperemia were unaffected by Finnish sauna bathing sessions of 10 and 20 min. These durations were chosen to reflect typical Finnish sauna bathing practice, whereby short (5–20 min) exposures are separated by rest or cooling periods outside of the sauna. The longer duration employed (2 × 10 min) reflects the duration (>19 min) associated with a reduced risk of cardiovascular mortality in middle‐aged Finnish men (Laukkanen et al. 2015). In addition, this duration is greater than the mean duration (~14 min) reported for the groups of regular Finnish sauna users (2–3 and 4–7 times per week) who displayed a reduced risk of cardiovascular mortality (Laukkanen et al. 2015). The lack of acute improvement in brachial artery flow‐mediated dilation and reactive hyperemia with the current protocol suggests that acute improvements in flow‐mediated dilation and reactive hyperemia may not contribute to the long‐term cardiovascular health benefits of Finnish sauna bathing. That said, we cannot rule out the possibility that Finnish sauna bathing sessions of longer duration acutely improve flow‐mediated dilation and reactive hyperemia. However, longer sessions may be difficult to tolerate, particularly in naïve sauna users. One limiting factor to increased sauna durations is the reduction in blood pressure elicited by Finnish sauna bathing. Indeed, blood pressure was significantly lowered during sauna exposure (Table 2), and one participant had to be removed from the sauna before the end of the second exposure during the 2 × 10 condition due to hypotension.

Previously observed improvements in flow‐mediated dilation and/or reactive hyperemia with passive heat exposure have been primarily ascribed to sustained increases in arterial shear stress, although increased expression of heat shock proteins may also contribute (Cheng and MacDonald 2019). In particular, passive heat exposure exposes the vascular endothelium to elevated levels of antegrade shear stress (Carter et al. 2014; Romero et al. 2017), a stimulus which induces an anti‐atherogenic profile (Chiu et al. 2009). However, this paradigm has been derived from experimental protocols that examined vascular adaptations following repeated (3–5 times/week for 8 weeks) exposures to relatively large heating stimuli (30–90 min of hot water immersion) (Carter et al. 2014; Brunt et al. 2016b). In contrast, the acute effect of passive heat exposure on flow‐mediated dilation and reactive hyperemia is variable (Cheng and MacDonald 2019). To date, six studies have examined the acute effect of passive heat exposure on flow‐mediated dilation and/or reactive hyperemia (Tinken et al. 2009; Brunt et al. 2016c; Ogoh et al. 2016; Thomas et al. 2016; Romero et al. 2017; Coombs et al. 2018). Of these studies, one observed an acute improvement in brachial artery flow‐mediated dilation in healthy young adults (Tinken et al. 2009), whereas another study observed an acute improvement in superficial femoral artery flow‐mediated dilation and peak reactive hyperemia in healthy older adults (Romero et al. 2017). Although mean skin temperature, oral temperature, and heart rate increased during both sauna conditions of the current study, it is possible that the heating stimulus was insufficient to induce an acute improvement in flow‐mediated dilation or reactive hyperemia. To our knowledge, no study has established whether a threshold level of shear stress and/or core temperature must be reached for passive heat exposure to acutely improve flow‐mediated dilation or reactive hyperemia. Within this context, our findings provide some perspective as to the doses of heating that might be insufficient to improve brachial artery flow‐mediated dilation and reactive hyperemia, at least in healthy middle‐aged and older adults. Regardless, it should be considered that the purpose of this study was not to induce a given level of thermal stress, but rather to determine if typical Finnish sauna bathing sessions (such as those associated with a reduced risk of cardiovascular mortality) elicit acute improvements in flow‐mediated dilation or reactive hyperemia.

As evidenced from the individual data (Fig. 1), there was variability in the flow‐mediated dilation response from pre‐ to postintervention. A pre‐ to postchange in unadjusted FMD greater than our a priori threshold of 1.5% was observed in 5 out of 16, 6 out of 16, and 5 out of 19 participants during the control, 1 × 10, and 2 × 10 conditions, respectively. Within the sauna conditions, there were no differences in sex or age between participants that demonstrated such an increase in flow‐mediated dilation relative to those who did not (data not reported). In the available duplicate (*n* = 4) or triplicate (*n* = 14) measurements of preintervention flow‐mediated dilation, the coefficient of variation for brachial artery diameter prior to cuff inflation was 4.1% and the intraclass correlation coefficient was 0.914.

Some limitations of this study warrant consideration. First, technical limitations prevented us from measuring brachial artery shear rate during the sauna exposures. The extent to which it increased during sauna bathing is therefore unknown. Second, we did not obtain a direct measure of internal body temperature. Oral temperature generally underestimates direct measures of internal body temperature, such as rectal and esophageal temperatures (Mazerolle et al. 2011). As such, it is possible that internal body temperature increased more than the 0.5–0.9°C increase in oral temperature observed during sauna bathing (Table 2). Indeed, it has previously been shown that esophageal temperature increases by ~1.4°C within ~9 min of Finnish sauna bathing (Kauppinen 1989b). Third, postsauna measurements were performed ~40 min after sauna bathing to ensure a return of brachial artery diameter to baseline values. We therefore cannot rule out that acute improvements in flow‐mediated dilation and/or reactive hyperemia may have been observed had we performed the measurements within a shorter time frame. To our knowledge, no study has examined the time course of vascular adaptations following passive heat exposure. Nonetheless, acute improvements in superficial femoral artery flow‐mediated dilation and peak reactive hyperemia have been observed when measurements are performed ~30 min following passive heat exposure (Romero et al. 2017). Third, we only examined the acute response to sauna bathing in healthy middle‐aged and older adults. Future studies are needed to determine if Finnish sauna bathing improves flow‐mediated dilation or reactive hyperemia in individuals with established vascular dysfunction, such as those with cardiovascular risk factors or overt cardiovascular disease. Fourth, we examined the acute effect of Finnish sauna bathing, which does not rule out the possibility that repeated or chronic Finnish sauna bathing leads to improved flow‐mediated dilation or reactive hyperemia.

In conclusion, this study tested the hypothesis that typical Finnish sauna bathing sessions acutely improve flow‐mediated dilation and reactive hyperemia in healthy middle‐aged and older adults. In contrast to our hypothesis, brachial artery flow‐mediated dilation and reactive hyperemia were unaffected by Finnish sauna bathing sessions of 10 and 20 min. These findings suggest that acute improvements in these markers of vascular health may not underlie the long‐term cardiovascular health benefits associated with Finnish sauna bathing in healthy middle‐aged and older adults.

## Conflict of Interest

None.
